# A Comparative Study Between Plate Fixation and Intramedullary Fixation for Displaced Mid-Shaft Clavicle Fractures in Adults

**DOI:** 10.5704/MOJ.2203.006

**Published:** 2022-03

**Authors:** A Vajrangi, SV Shetty, RM Shenoy

**Affiliations:** 1 Department of Orthopaedics, Jagadguru Jayadeva Murugarajendra (JJM) Medical College, Davangere, India; 2 Department of Orthopaedics, Yenepoya Medical College, Mangalore, India

**Keywords:** clavicle fracture, plate osteosyntheses, intramedullary nail fixation, DASH score

## Abstract

**Introduction:**

Clavicle fractures are frequently encountered by orthopaedic surgeons. Though multiple treatment techniques have been described in literature, open reduction and internal fixation with plating and intramedullary nailing are preferred in adults. This study analyses the functional outcome, complications, duration of bony union of mid-shaft clavicle fractures treated with plate fixation versus intramedullary fixation.

**Materials and methods:**

A quasi-experimental study was conducted on 38 patients with mid-shaft clavicle fracture aged between 18 to 60 years at a tertiary care centre in South India. Nineteen patients were treated by plate fixation and 19 patients underwent intramedullary nail fixation. Every alternate patient with mid-shaft fracture clavicle was treated with plate osteosyntheses or nailing.

**Results:**

The average age of presentation was 42 years in plate fixation and 31 years in nail fixation group. Robinson’s classification type 2B1 was the most common type of fracture in both groups. The Visual Analogue Scale (VAS) score reduced significantly from pre-operative to postoperative day 1 in both the groups but there was no significant difference between the two groups. The Disabilities of the Arm, Shoulder and Hand (DASH) score at 6, 12 and 24 weeks reduced significantly in both the groups but showed no statistically significant difference between the two groups. However, intramedullary nail fixation group had a shorter duration of hospital stay and showed an earlier improvement in the DASH score during the first six months after surgery. Complications included implant loosening (three patients) in plating group and implant failures (two cases) in nailing group.

**Conclusion:**

There is a role for surgical intervention for certain cases of displaced and comminuted clavicle fracture in order to avoid malunion or non-union and their potential long-term sequelae. Both the surgical modalities of plating, and nailing provide good functional outcome and allow early return to occupational activities.

## Introduction

The clavicle is one of the most frequently fractured bones^1^. The mid-shaft fracture of the clavicle is the most common, constituting up to 80% of the clavicular fractures as it is the thinnest part of the bone devoid of any muscular or ligamentous attachment^2,3^. Mid-shaft clavicular fractures have been classically managed conservatively, but due to unsatisfactory outcome in the form of non-union and symptomatic malunion, there has been a trend towards operative management^4-10^. Various modalities of fixations are available which include elastic stable intra-medullary nailing, intramedullary K-wires and plate fixation^2^. Optimal methods to treat displaced mid shaft clavicular fractures continue to be a topic of debate. Intramedullary fixation is a less invasive procedure with less blood loss and shorter duration of stay in the hospital. It has disadvantages like malrotation, overriding of fragments, irritation at the entry site, implant migration and needs implant removal. Plate osteosyntheses fixation is a more stable procedure with less chances of malrotation and overriding, but has disadvantages like bigger scar, prominence of plate leading to skin necrosis and infection^11^.

We conducted this study to compare the clavicle fractures treated by plate osteosyntheses and intramedullary fixation in terms of time for consolidated union, pain score, complications of each treatment modality, and functional outcome.

## Materials and Methods

We conducted a quasi-experimental study in a tertiary care centre in South India between November 2017 to October 2019 after obtaining institutional ethical committee clearance. Thirty-eight patients with fully displaced midshaft fracture of clavicle of less than 14 days duration and aged between 18 to 60 years requiring surgical intervention were included. Plain radiograph of the clavicle with shoulder was taken in anteroposterior view to assess the site of fracture and the type of fracture (displacement, angulation, comminution). The fractures were classified according to Robinson’s classification. Patients with mid-shaft fracture of clavicle as per Robinson’s classification type 2B1 and type 2B2 were enrolled into the study. Every alternate patient was treated with intramedullary nailing or plate osteosyntheses. The patients were explained in detail about the merits and demerits of the surgery (plate osteo syntheses or intra medullary nailing) and a written informed consent was taken. We excluded patients with open fractures, pathological fractures, other significant ipsilateral injuries of upper extremity, and head injury.

Patients undergoing plate osteosyntheses were placed in beach chair position. An incision was made over the clavicle following which supraclavicular nerves were identified and preserved. Fracture was reduced and fixed using a 3.5mm anatomical locking plate. Inter fragmentary screw was used wherever possible. Implant removal was not routinely performed except post union, in the presence of infection or on patient’s request.

In patients undergoing intramedullary nail fixation, an incision was made on the anterior aspect of the medial end of the clavicle. Entry was made using bone awl. A closed reduction of the fracture was performed and a titanium elastic nail (TENS nail) of appropriate diameter was inserted and passed across the fracture site to the lateral end. In some cases, open reduction was required. Implant removal was performed in all patients after union. No difficulties were encountered during the implant removal.

Pain was assessed using Visual Analog Scale (VAS) score on post-operative day 1. A follow-up visit was scheduled at 6 weeks, 12 weeks and 24 weeks after the surgery. Surgical outcome of both the methods was assessed in terms of time taken for union, functional outcome at each follow-up visit, pain scores and complications of each surgical method. Functional outcome was assessed using Disabilities of the Arm, Shoulder and Hand (DASH) score.

Data collected was analysed in SPSS version 22. Numerical continuous data were defined as mean ± standard deviations. Independent sample t test was used for comparing parametric data and Mann–Whitney U test for non-parametric data. Chisquare test or Fisher’s exact test were used for categorical comparison wherever appropriate. A p-value of less than 0.05 was considered to be statistically significant.

## Results

Our study included thirty-eight patients with displaced midshaft fracture of the clavicle. Nineteen patients were treated with open reduction and internal fixation using anatomical locking plate and screws, while the remaining nineteen patients were treated with closed/ open reduction and internal fixation using an intra medullary implant. No patients were lost for follow-up in both the groups. Most of the patients were above forty years in plating group (63.2%) while majority were less than forty years in nailing group (73.7%). The difference of the age between the groups was found to be significant (p=0.008). Majority of the patients in both groups were males. The most common mechanism of injury was fall on an outstretched hand which accounted for nearly 52.6% of cases in plating group and 63.2% of cases in intramedullary nailing group (Table I).

**Table I:** Baseline characteristics of both the groups

Characteristics	Plating (n=19)	Nailing (n=19)	P value
Mean Age ± SD in years	42.74 ± 14.05	31.32 ± 10.58	0.008*
Male : Female	14 : 5	16 : 3	0.612
Right : Left	8 : 11	13 : 6	0.587
Mean Interval from injury to surgery in days ± SD	3.11 ± 1.41	3.16 ± 1.68	0.917
Mode of injury			
RTA	5	4	-
Fall on outstretched hand	10	12	-
Fall on shoulder	3	1	-
Sports injury	-	1	-
Others	1	1	-
Robinson’s classification			
Type 2B1	15	12	-
Type 2B2	4	7	-

The mean functional DASH score for plating and nailing groups pre-operatively was 85.64 and 86.13, respectively. It was then re-assessed at 6 weeks, 12 weeks, and 24 weeks. At the end of 24 weeks, DASH score reduced significantly in both the study groups i.e. 12.32 for plating and 11.45 for nailing group. This suggested a good functional outcome in both groups at the end of 24 weeks. But no statistically significant difference was noted in the functional outcome between the two groups (Table II). Mean VAS scores for pain reduced significantly from pre-operative to 1st postoperative day from 9.705 to 5.384 and from 9.76 to 5.43 in plating and nailing groups, respectively (p value <0.001). But there was no statistical significance in the difference between VAS score on post-operative day one between the two groups (p=0.876).

**Table II:** Functional DASH score, VAS score for pain on 1st post-operative day and time taken for union in both the groups

Parameters	Plating (n=19) Mean± SD	Nailing(n=19) Mean± SD	p value
DASH Score pre-operative	85.64±3.308	86.13±3.341	0.715
DASH Score at 6 weeks	61.005±8.115	59.837±6.975	0.637
DASH Score at 12 weeks	43.068±8.28	41.095±9.67	0.504
DASH Score at 24 weeks	12.316±6.909	11.45±4.816	0.658
VAS score pre-op	9.705±0.513	9.76±0.373	0.693
VAS score on 1st post-op day	5.3842±0.836	5.43±1.014	0.876
Time for union in weeks	12.89±3.234	12.67±1.534	0.675
Mean duration of hospital stay in days	9.74±2.182	7.95 ± 3.118	0.048

The hospital stay for nailing group (mean 7.95 days) was shorter than that for plating group (mean 9.74 days). This difference was statistically significant (p=0.048). The difference in time taken for union in weeks between the plating group (12.89 with SD of 3.23) and nailing group (12.67 with SD of 1.53) was statistically not significant (p=0.675, Table II)

**Fig. 1:**
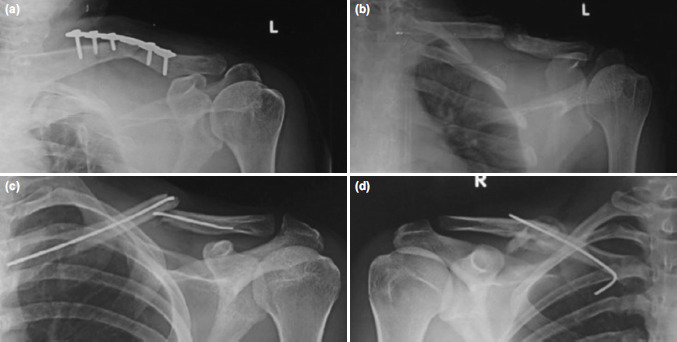
Complications following plating, (a) Implant loosening and backing out of screws, (b) Non-union of the fracture post implant removal; complications following nailing, (c) Implant failure following re-fracture. (d) Implant failure due to piercing of the nail through comminuted fragments dorsally.

Complications were seen in six patients (31.5%) in each group (Table III). Three patients (15.8%) developed implant loosening (Fig 1a) in plating group. They also had restriction of movement of the shoulder as they were immobilised for longer periods. One of these cases (5.3%) led to non-union (Fig 1b). One patient (5.3%) had prominence of plate with irritation of skin. Four patients (21.05%) required implant removal out of which three had implant loosening and one had plate prominence. Implant removal was also done in six more patients on their request post union after one year of surgery.

**Table III:** Complications following plating and nailing

Complication	Plating Number (Percentage)	Nailing Number (percentage)
Superficial infection	-	1 (5.3)
Implant irritation	1 (5.3)	3 (15.8)
Implant failure	-	2 (10.5)
Implant loosening	3 (15.8)	-
Non-union	1 (5.3)	2 (10.5)
Mal-union	-	-
Delayed union	1 (5.3)	-
Restriction of movement	3 (15.8)	-

In the nailing group, one patient (5.3%) had superficial infection on the medial side at point of entry of nail. Three patients (15.8%) had irritation of the skin on the medial end of clavicle from where the nail was inserted. Implant removal was performed in all the patients after six months of surgery. No difficulties were encountered during the implant removal in both the groups. Two patients (10.5%) had implant failure and non-union of which one re-fractured due to a fall (Fig 1c) and the other had migration of implant through the comminuted fragment (Fig 1d). There was no statistically significant difference on comparing the complications in both the groups (p=0.189) (Table III).

## DISCUSSION

In our study, we compared the results of thirty-eight patients treated with plating (n=19) and nailing (n=19). Our study showed a male preponderance in both the groups. All the previous studies have concluded that incidence of clavicular fractures is significantly more common in males due to high energy trauma^12-15^. We noted that the plating group had more patients above 40 years of age as compared to the nailing group. Robinson 2B1 type of mid shaft clavicular fracture was the most common type in our study as well as other studies^12,13^.

The duration of hospital stay in intra medullary nail group was significantly lower when compared to that of plate group which is likely to be due to shorter operation period and faster recovery. The same results were noted in other studies^12,13^.

We used DASH score to assess the functional outcome. There was significant improvement in functional outcome in each group but no significant difference between the two groups at the end of 24 weeks. Studies conducted by Andrade-Silva *et al*^12^ and Meijden *et al*^13^ also found no significant difference in the functional outcome in the results of plating versus nailing. The VAS score on post-op day 1 was 5.3842 ± 0.836 for plating and 5.43 ± 1.014 for nailing, which was not significant. Silva *et al* also used VAS score to compare between plating and nailing and found no significance^12^. We considered the fracture to be united when there was no tenderness clinically, the fracture line was not visible radiologically, and full unprotected function of the limb was possible. The time for the fracture union has been variable in the literature. In our study, the plating group required 12.89 ± 3.2 weeks and nailing group required 12.67 ± 1.5 weeks for bony union. This timeline was comparable with the study conducted by Zehir *et al* when the time required for bony union in the plating group and nailing group were 13.81 ± 2.9 weeks and 11.75 ± 2.7 weeks, respectively^16^. There was no significant difference in the time taken for union between the two groups in our study as well as other studies^16^.

In our study, major complications in plating group were implant loosening (three patients) of which one patient developed non-union. In the nailing group, the major complication was implant failure and non-union (two patients). There was no statistical significance in the number of complications between the two groups (p=0.189). A study conducted by Meijden *et al* showed no statistical significance in the complications between plating and nailing groups^13^. In the study done by Silva *et al*, there was one major complication of non-union in nailing group with no major complications seen in plating group. They found a significant difference in implant related pain between two groups. Implant related pain was seen in 40% of cases in nailing group while plating group had only 14% of implant related pain^12^. Table IV summarises the results of our study in comparison with previous studies.

**Table IV:** Comparison of our study findings with previous studies

Characteristic	Our study (n=38)		Andrade-Silva *et al* (n=59)		Van de Meijden *et al* (n=120)	
	Plate Fixation	Intra medullary Fixation	Plate Fixation	Intra medullary Fixation	Plate Fixation	Intra medullary Fixation
No. of patients	19	19	33	26	58	62
Mean age (years) ± SD	42.74±14.05	31.32±10.58	31.2±12.1	28.3±9.4	38.4±14.6	39.6±13.2
Male : Female ratio	14:5	16:3	28:5	19:7	53:5	60:2
DASH score at 6 months	12.315±6.909	11.45±4.816	9.9±10.9	8.5±13.0	3	5.6
VAS Score on 1st pre-operative day	5.3842±0.836	5.43±1.014	4.1±2.5	3.8±3.0	NR	NR
Time to union in weeks	12.89±3.234	12.67±1.534	16.8±3.5	15.9±3.5	NR	NR
**Complications**						
Infection (superficial)	NR	1	NR	NR	NR	NR
Hypertrophic scar	1	NR	NR	NR	NR	NR
Implant loosening	3	NR	NR	NR	NR	NR
Implant prominence and irritation	1	3	4	10	2	NR
Implant failure	NR	2	10	1	1	1
Restriction of movements	3	NR	NR	NR	NR	NR
Non-union	1	NR	NR	1	1	NR
Mal-union	NR	NR	NR	NR	NR	NR

Abbreviation – NR: Not reported

The optimal method of fixation whether plate or nail is still debatable. However current studies do not show any difference in both modalities^3^. The loosening of implants in three cases of plating group delayed the mobilisation of shoulder joint which led to restriction of shoulder movements in our study. We observed that there was no interfragmentary screw used in these cases to stabilise the fractures probably suggesting that interfragmentary screws wherever possible provides better bone to bone contact. The other probable reasons for implant loosening in the plating group could be early lifting of weights, latent infection, and improper technique of fixation. Of the two patients in the nailing group with implant failure, one had refracture following trauma and the other patient had comminuted fracture of mid- shaft clavicle. This probably suggests that opting for nailing in this fracture was not a right choice of fixation. The disadvantage of nailing observed was the irritation caused at the skin on the medial point of entry^12^. Three patients complained of irritation and pain at the point of entry of the skin, as the end of the titanium elastic nail was remained subcutaneously. The main disadvantage of elastic stable intra medullary nailing is the projection of implant causing irritation and pain. Technical recommendations suggested, cutting the nail close to the bone cortex with bending of the tip and the use of a medial end cap might theoretically prevent nail protrusion but need to be studied further and remains as an important issue to be addressed when this method is employed^12,14^. Literature has shown rotational malalignment of fractures happening when nailing was used^11^. In our study, contrary to this no malrotations were observed in the nailing group. The application of bone graft in comminuted fracture is recommended but using it primarily is anecdotal^12^. There was only one case of nonunion in plating group suggesting that surgical treatment had better outcome than conservative treatment^2^. There were no catastrophic neurovascular complications in the operative group. Patients were satisfied with the shoulder and its appearance following the operative intervention.

Intramedullary nail fixation group had good functional outcome during the first six months after surgery with early functional recovery, and lesser duration of stay in the hospital. Intramedullary nail fixation group has the advantage of being less invasive surgery as it is performed with a small incision.

## Conclusion

There is a role for surgical intervention for certain cases of displaced and comminuted clavicle fracture in order to avoid malunion or non-union and their potential long-term sequelae. Both the procedures, plate fixation and intra medullary nail fixation provide good anatomic reduction, stable fixation, early return to occupational activities and satisfying functional results. There was no significant difference in functional result, time for union and complications in both groups.
